# Cold or calculating? Reduced activity in the subgenual cingulate cortex reflects decreased emotional aversion to harming in counterintuitive utilitarian judgment

**DOI:** 10.1016/j.cognition.2012.11.002

**Published:** 2013-03

**Authors:** Katja Wiech, Guy Kahane, Nicholas Shackel, Miguel Farias, Julian Savulescu, Irene Tracey

**Affiliations:** a Nuffield Department of Clinical Neurosciences, Nuffield Division Anaesthetics, University of Oxford, John Radcliffe Hospital, Headley Way, Oxford OX3 9DU, UK; b Oxford Centre for Functional Magnetic Resonance Imaging of the Brain, Nuffield Department of Clinical Neurosciences, University of Oxford, John Radcliffe Hospital, Headley Way, Oxford OX3 9DU, UK; c Oxford Centre for Neuroethics, University of Oxford, Littlegate House, St. Ebbes Street, Oxford OX1 1PT, UK; d Oxford Uehiro Centre for Practical Ethics, University of Oxford, Littlegate House, St. Ebbe’s St., Oxford OX1 1PT, UK; e Department of Philosophy, University of Cardiff, UK; f Department of Experimental Psychology, University of Oxford, South Parks Road, Oxford OX1 3UD, UK

**Keywords:** ACC, anterior cingulate cortex, DLPFC, dorsolateral prefrontal cortex, EPI, echo-planar imaging, EPQ-R, Eysenck Personality Questionnaire (revised), FWHM, full-width at half maximum, fMRI, functional magnetic resonance imaging, MNI, Montréal Neurological Institute, MR, magnetic resonance, MRI, magnetic resonance imaging, SCC, subgenual cingulate cortex, SPM, statistical parametric mapping, VMPFC, ventromedial prefrontal cortex, Moral decision-making, Cingulate cortex, Utilitarian judgment, Functional magnetic resonance imaging, Social cognition

## Abstract

Recent research on moral decision-making has suggested that many common moral judgments are based on immediate intuitions. However, some individuals arrive at highly counterintuitive utilitarian conclusions about when it is permissible to harm other individuals. Such utilitarian judgments have been attributed to effortful reasoning that has overcome our natural emotional aversion to harming others. Recent studies, however, suggest that such utilitarian judgments might also result from a decreased aversion to harming others, due to a deficit in empathic concern and social emotion. The present study investigated the neural basis of such indifference to harming using functional neuroimaging during engagement in moral dilemmas. A tendency to counterintuitive utilitarian judgment was associated both with ‘psychoticism’, a trait associated with a lack of empathic concern and antisocial tendencies, and with ‘need for cognition’, a trait reflecting preference for effortful cognition. Importantly, only psychoticism was also negatively correlated with activation in the subgenual cingulate cortex (SCC), a brain area implicated in empathic concern and social emotions such as guilt, during counterintuitive utilitarian judgments. Our findings suggest that when individuals reach highly counterintuitive utilitarian conclusions, this need not reflect greater engagement in explicit moral deliberation. It may rather reflect a lack of empathic concern, and diminished aversion to harming others.

## Highlights

► We investigated the role of two personality traits in moral judgment using fMRI. ► Psychoticism and need for cognition correlated with number of utilitarian decisions. ► Only psychoticism was associated with reduced activation in the subgenual ACC. ► Our data suggest that utilitarian judgments can reflect a reduced aversion to harm. ► They offer little support to a tie between utilitarian judgment and greater deliberation.

## 1 Introduction

Most people think that it would be wrong to kill a stranger by pushing him onto the track of a runaway trolley in order to save the lives of five others. There is, however, a small minority that adopts the utilitarian view that we *should* push the stranger because this would save a greater number of lives (Cushman, Young, & Hauser, 2006). Such utilitarian views are controversial. Many people find them repugnant, and utilitarianism is often portrayed as a cold and calculating outlook that is due to a deficient affective sensibility (Hazlitt, 1824/1991). Utilitarians, however, argue that their counterintuitive conclusions are simply what results when, instead of just following our immediate gut reactions, we use moral reasoning to critically scrutinize them (Singer, 2005; Unger, 1996). They claim that utilitarians are not colder than other people; but they may appear so because they are more ‘calculating’ or rational.

Recent research has been taken to support for the latter view. Neuroimaging studies of affect-laden moral dilemmas have been taken to suggest that non-utilitarian or ‘deontological’ judgments (e.g. ‘don’t push the stranger’) are based on a pre-potent emotional aversion to directly harming others (Greene, Nystrom, Engell, Darley, & Cohen, 2004; Greene, Sommerville, Nystrom, Darley, & Cohen, 2001). By contrast, utilitarian judgments (e.g. ‘push the stranger to save five others’) in difficult dilemmas were associated with increased activation in the dorsolateral prefrontal cortex (DLPFC) and inferior parietal lobe, areas implicated in deliberative processing, and in the dorsal anterior cingulate cortex (dACC), an area implicated in the detection and resolution of conflict (Greene et al., 2004). In addition, recent studies have reported that cognitive load increased response times in utilitarian judgments but not in deontological ones (Greene, Morelli, Lowenberg, Nystrom, & Cohen, 2008), and that subjects higher on ‘need for cognition’ (Cacioppo, Petty, Feinstein, & Jarvis, 1996), a motivational tendency to seek and enjoy effortful cognitive activity, exhibited greater rates of utilitarian judgment (Bartels, 2008). These findings suggest that utilitarian judgments involve the use of effortful, deliberative processing to overcome an immediate emotional response (Greene, 2008; though see Kahane, 2012; Kahane & Shackel, 2010; Kahane et al., 2012).

Several recent studies, however, indicate that utilitarian judgment can also be the consequence of a lack of empathic concern. Some evidence for this comes from clinical populations. Patients with lesions in the VMPFC (Ciaramelli, Muccioli, Làdavas, & di Pellegrino, 2007; Koenigs et al., 2007; Moretto, Làdavas, Mattioli, & di Pellegrino, 2009) and with frontotemporal dementia (Mendez, Anderson, & Shapira, 2005), conditions associated with deficits in empathic concern and social emotion and with disordered social behavior, exhibit increased rates of utilitarian judgment in emotionally-loaded moral dilemmas, apparently because such patients lack the prepotent aversive response to harming. A recent study has shown that utilitarian judgments in patients with VMPFC damage were associated with weaker skin conductance responses, and with shorter reaction times, compared to healthy subjects (Moretto et al., 2009), further suggesting that in these patients utilitarian judgments do not require the overcoming of an aversion to harming others.

However, diminished social emotion can also be found in the non-clinical population. It is thus plausible that utilitarian judgments in healthy individuals might also be rooted in an atypically weak or even absent aversion to harming others. This would explain the otherwise puzzling findings that increased rates of utilitarian judgment in healthy individuals are predicted by individual differences in aversive reactivity to harming others, as indexed by peripheral vasoconstriction (Cushman, Gray, Gaffey, & Mendes, 2012), and are associated with lower response times (Greene et al., 2008) and reduced skin conductance response (Moretto et al., 2009). In addition, recent studies report that such a tendency to utilitarian judgment in healthy subjects is associated with lower rates of trait empathy (Choe & Min, 2011; Crockett, Clark, Hauser, & Robbins, 2010), and higher levels of testosterone (Carney & Mason, 2010), which has been associated with reduced empathic concern (Hermans, Putman, & Van Honk, 2006). Most importantly, several recent studies report greater rates of utilitarian judgment in individuals high on psychopathy (Bartels & Pizarro, 2011; Glenn, Koleva, Iyer, Graham, & Ditto, 2010; Koenigs, Kruepke, Zeier, & Newman, 2012), although interestingly such a relation was not observed in some studies of psychiatric patients and criminal offenders (Cima, Tonnaer, & Hauser, 2010; Glenn, Raine, & Schug, 2009).

There is thus a growing body of evidence indicating that utilitarian judgment in the healthy population may be based, not in greater deliberative effort as suggested by earlier research (Greene, 2008), but in a diminished or absent aversion to harming that is, moreover, associated with antisocial traits (Bartels & Pizarro, 2011). On this emerging picture of utilitarian judgment, utilitarians approach moral decisions in a calculating manner *because* they are ‘colder’ than other people.^2^

However, the neural mechanisms that underlie individual differences in utilitarian judgment remain unclear, and there has so far been no attempt to integrate these seemingly contrasting lines of evidence. One plausible hypothesis is that there are two distinct pathways to utilitarian judgement. Some individuals might make utilitarian judgments because they are more calculating or ‘rational’, and others because they are colder. It cannot yet be ruled out, however, that the evidence associating utilitarian judgment with greater cognitive effort at least partly reflects the more calculating form that moral decision-making takes in the absence of normal emotional input.

Here, we used functional magnetic resonance imaging (fMRI) in healthy volunteers to investigate the neural basis of reduced aversion to harming in counterintuitive utilitarian judgment. Unlike previous studies, we employed measures of individual differences both in ‘coldness’ and in ‘calculation’. We predicted that two distinct personality traits would be associated with a greater tendency to utilitarian judgment in emotionally-loaded dilemmas: *need for cognition*, a motivational tendency to seek effortful cognitive activity (Cacioppo et al., 1996), and *psychoticism*, a subscale of the Eysenck Personality Questionnaire (Eysenck & Eysenck, 1991) that reflects lack of emotionality, diminished empathic concern, aggression and non-conformity to social norms. Psychoticism has been associated with reduced sensitivity to violence in normal individuals (Bruggemann & Barry, 2002) as well as with psychopathy (Hare, 1982; Shine & Hobson, 1997), a construct often seen as lying on a continuum with psychoticism (Corr, 2010; Eysenck, 1992) although, unlike psychopathy, psychoticism primarily aims to capture a general dimension of personality in the normal population.

We recently showed that counterintuitive judgments across a range of moral contexts are associated with greater perceived difficulty and with activation in the subgenual part of the anterior cingulate cortex (Kahane et al., 2012), a brain area previously implicated in the processing of social information (Behrens, Hunt, Woolrich, & Rushworth, 2008), and in altruistic donation decisions (Moll et al., 2006). The subgenual cingulate cortex (SCC) has been repeatedly implicated in negative emotions such as sadness (Mayberg et al., 2000), including feeling sad for the victim of a harmful action (Decety, Michalska, & Kinzler, 2012). Several recent studies have also associated the SCC with guilt, a self-directed negative emotional response to the belief that one has violated a moral norm (Green, Ralph, Moll, Stamatakis, & Zhan, 2010; Zahn, de Oliveira-Souza, Bramati, Garrido, & Moll, 2009b; Zahn et al., 2009a). Importantly, SCC activation in the context of guilt was found to reflect individual differences in empathic concern (Zahn et al., 2009b).

Interestingly, the lesions in patients with VMPFC damage typically extend to the SCC, and previous studies report that individuals with VMPFC damage are significantly less likely to feel guilt when they violate social norms (Krajbich, Adolphs, Tranel, Denburg, & Camerer, 2009). Indeed, it has been suggested that VMPFC patients exhibit greater rates of utilitarian judgment due to a deficit in empathic concern and the capacity to feel guilt (Moll & de Oliveira-Souza, 2007; Moretto et al., 2009). In line with this, a recent study found that healthy individuals predominantly associate utilitarian judgments with feeling guilty (Choe & Min, 2011).

These previous findings suggest that activation in the SCC may reflect aversive feelings such as guilt that are associated with agreeing to harm others and generally with the perceived violation of moral norms. Since psychoticism is associated with reduced empathic concern, aggression and insensitivity to social norms, we therefore predicted that utilitarian judgments by individuals higher on psychoticism should be reflected in decreased activation in the SCC yet not with increased activity in the DLPFC, a brain area generally implicated in explicit deliberation, and which has been previously associated with counterintuitive utilitarian judgment (Greene et al., 2004)—though as noted above, a reduced emotional response might also encourage a more ‘calculating’ approach to moral dilemmas. At the behavioural level, we predicted that such judgments would be associated with lower perceived difficulty.

By contrast, if utilitarian judgments by individuals higher on ‘need for cognition’ involve greater engagement of explicit deliberation, they should be associated with increased activation in the DLPFC but not with decreased activation in the SCC. In addition, we would expect that increased deliberative effort would be reflected in increase in perceived difficulty.

The above predictions are specific to moral dilemmas where, in order to save the greater number of lives, it is necessary to directly kill or significantly harm someone. In this type of dilemma, such a utilitarian choice is typically highly counterintuitive. Here, we also employed a range of new dilemmas that involved more everyday choices such as whether to lie to someone to avoid hurting them (see also Kahane et al., 2012). In these dilemmas, it is rather the deontological choice (e.g. to tell a harmful truth) that most people find counterintuitive. This new category of dilemma allowed us to disentangle the respective contribution of intuitiveness and content (utilitarian vs. deontological) to moral judgments. In order to investigate whether the hypothesized influence of ‘need for cognition’ and psychoticism on moral decision-making is specific to counterintuitive utilitarian judgment, we further examined their effect in this new category of dilemmas.

Although individuals higher on psychoticism might feel less bound by moral rules such as the prohibition against lying, they might also be less reluctant to bluntly assert deeply hurtful truths. It is thus unclear that psychoticism should be associated with a distinctive pattern of response to this type of dilemma. By contrast, one would expect individuals who are attracted to effortful reflection to exhibit a general tendency to arrive at moral conclusions that go contrary to common intuitions. We therefore predicted that ‘need for cognition’ would be associated with greater rates of counterintuitive judgment and DLPFC activation across both categories of moral dilemmas.

## 2 Method

The experimental setup and stimuli used in this study have been described elsewhere (Kahane et al., 2012). Here, we briefly summarize the methods and data analysis relevant to the research question of the present paper.

### 2.1 Subjects

Sixteen healthy, right-handed subjects (9 female, mean age, 29.25, range, 21–41) participated in the study. The volunteers were pre-assessed to exclude those who self-reported a previous history of neurological or psychiatric illness. All subjects gave informed consent, and the study was approved by the local Research Ethics Committee.

### 2.2 Experimental design and protocol

Two types of dilemmas were employed in the study: dilemmas where the deontological judgment is more intuitive (*DI dilemmas*^3^; *N* = 8), which were largely drawn from the ‘personal dilemmas’ used by Greene et al. (2001), as well as new dilemmas where the utilitarian judgment is more intuitive (*UI dilemmas*^4^; *N* = 10). ‘Utilitarian judgment’ was defined as the choice, of the two available, that would lead to the better aggregate consequence. A choice was classified as ‘intuitive’ (relative to a population) if it was chosen by 12 or more of 18 independent judges who were asked to give their unreflective opinion; the contrary choice was classified as ‘counterintuitive’ (for further details on the classification of dilemmas see Kahane et al., 2012).

The experiment participants performed while they were lying in the scanner was divided into four sessions, each lasting about 10 min. UI and DI dilemmas were presented in randomized order. Each dilemma was presented as text through a series of three screens, the first two describing a scenario and the last posing a question about the moral appropriateness of an action one might perform in that scenario (e.g., to push the large stranger). Each screen was presented for 15 s. After reading the third screen, subjects responded by pressing one of two buttons (“yes” or “no”) to indicate whether they agreed to the suggested solution. Participants were instructed to read the text quietly, and to press the response button as soon as they had made their decision. No visual feedback was given upon the decision. At the end of each dilemma subjects were prompted to rate the difficulty of the dilemma using a Numerical Rating Scale ranging from 0 (=“not difficult at all”) to 100 (=“very difficult”). Participants were given 6 s for the rating. At the end of each trial subjects were instructed to fixate a white cross that was displayed in the centre of the computer screen for 12 s (baseline).

### 2.3 Questionnaires

Prior to the scanning session all participants filled in paper-and-pencil versions of the Eysenck Personality Questionnaire (48-item EPQ-R Short Scale; Eysenck & Eysenck, 1991) and the short form of the ‘Need for cognition’ scale (Cacioppo et al., 1996).

### 2.4 Image acquisition

MR scanning was performed on a 3T MRI system (Oxford Magnet Technology, Oxford, UK) with the use of a Nova Medical quadrature birdcage coil (Nova Medical, Wilmington, USA). For the functional measurements, 33 axial slices (slice thickness 3 mm) were acquired using a gradient echo echo-planar (EPI) T2^*^-sensitive sequence (repetition time, 2.38 s, echo time, 30 ms; flip angle, 90°; matrix, 64 × 64; field of view, 192×192 mm^2^). Subjects wore MR-compatible electrostatic headphones to attenuate the scanner noise. For information regarding coverage of relevant brain regions, see Supplementary Figs. 1 and 2.

### 2.5 Data analysis

For the two personality traits (i.e., psychoticism and ‘need for cognition’) scores were calculated according to the questionnaires’ manuals and *z*-transformed to allow for direct comparison. Pearson correlation coefficients (1-tailed tests) were calculated for the relationship between the two questionnaire scores and (i) the number of counterintuitive utilitarian judgments (in DI dilemmas), (ii) the number of counterintuitive deontological judgments (in UI dilemmas), (iii) the average difficulty rating for counterintuitive utilitarian judgment and (iii) the average difficulty rating for counterintuitive deontological judgments.

For the neuroimaging data image preprocessing and statistical analysis were carried out using SPM5 (http://www.fil.ion.ucl.ac.uk/spm). The first five image volumes of each session were discarded to account for T1 relaxation effects. The remaining volumes were realigned to the sixth volume to correct for head motion before statistical analysis. The EPI images were spatially normalized (Friston et al., 1995) to the template of the Montréal Neurological Institute (MNI; Evans et al., 1993). The normalized EPI-images were smoothed using an 8-mm FWHM (full-width at half maximum) Gaussian kernel, temporally high-pass filtered (cut-off 128 s) and corrected for temporal autocorrelations using first-order autoregressive modelling.

In order to investigate the relationship between the two personality traits and the neural processes in the SCC and DLPFC during counterintuitive utilitarian judgments in DI dilemmas, we first computed two simple regression analyses. In the first simple regression analysis, first level images reflecting brain activation during counterintuitive utilitarian moral judgements were entered into a second level regression analysis with *z*-transformed psychoticism scores as a regressor. In the second analysis, individual ‘need for cognition’ scores were considered as a regressor in the analysis of brain responses during counterintuitive utilitarian judgments, again using first level images reflecting brain activation during counterintuitive utilitarian moral judgements. In order to identify brain regions showing a significantly stronger correlation with one of the two personality measures during counterintuitive utilitarian judgments, we subsequently performed a multiple regression analysis (Nichols, 2008). In this analysis, first level contrast images representing activation during counterintuitive utilitarian moral judgments were entered into a new model with the two questionnaire scores as covariates. The design matrix therefore comprised two regressors, one representing activation during counterintuitive utilitarian judgments that is correlated with individual psychoticism scores and one representing the same activation during counterintuitive utilitarian judgments but this time correlated with ‘need for cognition’ scores. On the group level, we used a “1 −1” contrast to test for any voxels showing a significantly stronger negative correlation with ‘need for cognition’ than with psychoticism using a *t*-statistic. Conversely, the contrast defined as “−1 1” tested for voxels showing a stronger negative correlation with psychoticism than for ‘need for cognition’.

In order to explore the results of the multiple regression analyses in more detail, parameter estimates were extracted from the peak voxel and plotted against the individual psychoticism and ‘need for cognition’ scores. Following a similar procedure recently adopted by Zahn et al. (2009b), we considered effects of the three regression analyses as significant if they survived an uncorrected voxel-level significance threshold of *p* < 0.01 and in addition a family-wise error (FWE) corrected threshold in a 5 mm sphere around the original left or right hemispheric homologue of the mean coordinate (Montreal Neurological Institute (MNI) coordinates (*x*, *y*, *z*): −8.5, 25.5, −5.5) of SCC activations that have recently been reported in relation to guilt and empathic concern (Moll et al., 2006; Zahn et al., 2009a). For the DLPFC, the same small volume correction was applied to a 5 mm sphere around the MNI coordinate (*x*, *y*, *z* = 16, 55, 8) reported by Greene et al., 2004 (though for a different anatomical labeling, see Moll & de Oliveira-Souza, 2007). All coordinates are given in MNI space.

Because neither of the two personality traits showed a significant correlation with the number of counterintuitive deontological decisions in UI dilemmas (see Results for details), we decided to not pursue the analysis of individual differences in counterintuitive deontological judgments at the neural level.

## 3 Results

### 3.1 Behavioral data

#### 3.1.1 Correlations between personality traits and number of counterintuitive utilitarian judgments

**Fig. 1:**
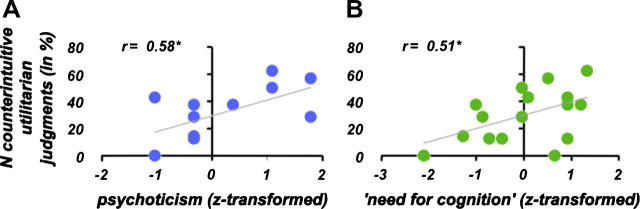
Correlation between personality traits and number of counterintuitive utilitarian judgments. (A) Psychoticism and (B) ‘need for cognition’ showed a significant positive correlation with the number of counterintuitive utilitarian judgments.

As predicted, the number of counterintuitive utilitarian decisions was positively correlated both with psychoticism (*r* = 0.58, *p* = 0.012; Fig. 1A) and with ‘need for cognition’ (*r* = 0.51, *p* = 0.023; Fig. 1B). These two scales were statistically independent (*r* = 0.07, *p* = 0.81).

The frequency of making a counterintuitive deontological decision was not significantly correlated with psychoticism (*r* = 0.10, *p* = 0.361) or ‘need for cognition’ (*r* = −0.33, *p* = 0.104).

#### 3.1.2 Correlations between personality traits and perceived difficulty

Based on our hypothesis that ‘need for cognition’ but not psychoticism should be associated with greater deliberative effort while making a counterintuitive decision, we investigated the correlation between the two personality traits and perceived difficulty during decision-making. For counterintuitive utilitarian decisions, ‘need for cognition’ scores showed a clear trend towards a positive correlation with perceived difficulty (*r* = 0.449, *p* = 0.05) while psychoticism (*r* = 0.029, *p* = 0.46) was not significantly correlated with difficulty ratings.

For counterintuitive deontological decisions, neither psychoticism nor ‘need for cognition’ showed a significant correlation with perceived difficulty (psychoticism: *r* = 0.434, *p* = 0.061; ‘need for cognition’: *r* = 0.133, *p* = 0.333). Given the observation that psychoticism and ‘need for cognition’ were not related to the number of counterintuitive deontological decisions or the perceived difficulty of making such judgment, we decided to not pursue the analysis of individual differences in counterintuitive deontological judgments at the neural level.

### 3.2 Neuroimaging data

**Fig. 2:**
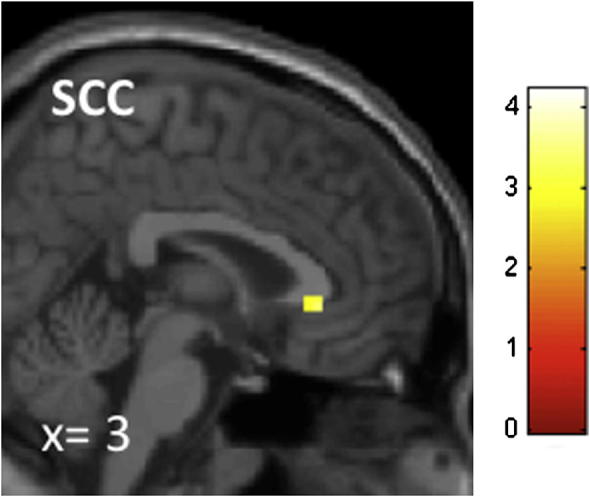
Activation in the SCC showing a negative correlation with ‘psychoticism’ scores during counterintuitive utilitarian judgment. The higher participants scored on the ‘psychoticism’ scale, the lower the activation level in the SCC during counterintuitive utilitarian judgment. Significant voxels are overlaid onto a standard structural scan in MNI152 space.

Because psychoticism but not ‘need for cognition’ should be associated with a diminished empathic concern and aversion to harming, only the first should show a negative correlation with SCC activation. Results of the two simple regression analyses confirm this hypothesis. The higher the participants scored on the psychoticism scale, the lower the activation in the SCC (*x*, *y*, *z* = 3, 30, −3; SVC: *x*, *y*, *z* = 9, 24, −3, *p* = 0.02, FWE-corrected) during counterintuitive utilitarian judgments (Fig. 2A; Table S1). A negative correlation with DLPFC activation did not survive correction. ‘Need for cognition’ showed no significant negative correlation with either of the two brain regions (Table S2).

**Fig. 3:**
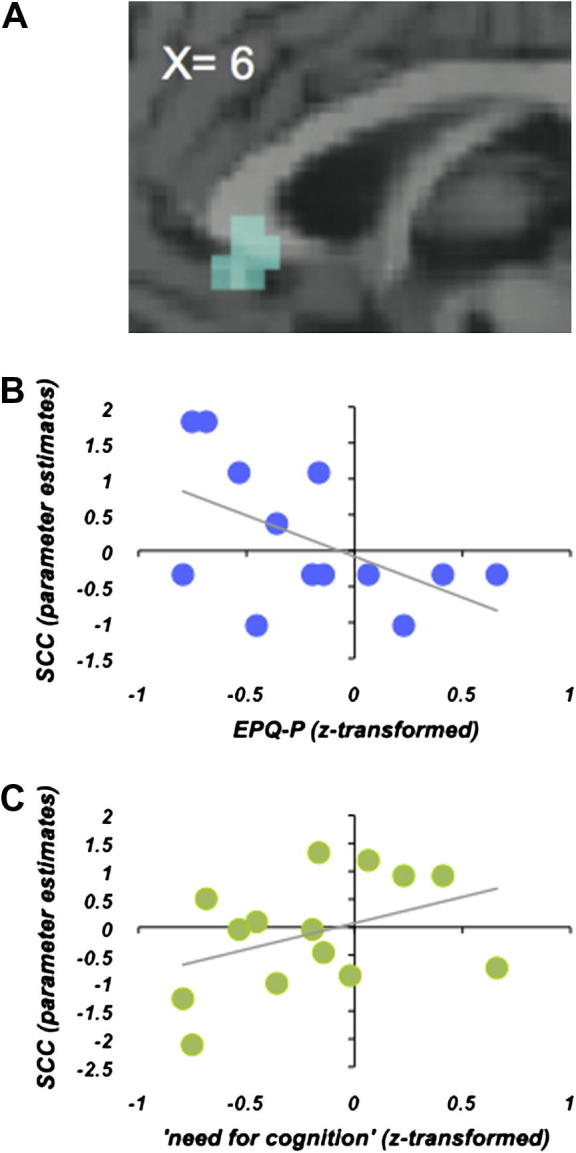
Correlation between personality traits and SCC activation during counterintuitive utilitarian judgement (multiple regression). (A) SCC cluster activated during counterintuitive utilitarian judgements showing a stronger negative correlation with psychoticism than with ‘need for cognition’. (B) Scatterplot of activation in the SCC (*x*, *y*, *z* = 6, 24, −3) illustrating the significantly negative correlation with psychoticism and (C) the non-significant positive correlation with ‘need for cognition’ during counterintuitive utilitarian judgements.

In a second step we directly compared the correlations with the two personality traits using a multiple regression analysis. In this analysis, first level contrast images representing activation during counterintuitive utilitarian moral judgments were entered into a new model with the two questionnaire scores as covariates. The direct comparison of the two covariate regressors revealed that activation in the SCC was differentially correlated with psychoticism and ‘need for cognition’ (*x*, *y*, *z* = 6, 24, −3; SVC: *x*, *y*, *z* = 9, 24, −3, *p* = 0.036, FWE-corrected; Fig. 3A; Table S3). In order to explore this difference in more detail, parameter estimates for activation corrected with psychoticsm and ‘need for cognition’ were extracted and plotted against the individual scores of both questionnaires. As shown in Fig. 3B and C, psychoticism showed a significantly negative correlation with SCC activation during counterintuitive utilitarian decisions while ‘need for cognition’ was positively correlated with SCC activity (albeit below statistical significance). Activation in the DLPFC was not differentially correlated with the two personality traits.

## 4 Discussion

Some individuals reach utilitarian conclusions that are contrary to widely held moral views and intuitions. One way to arrive at such nonconformist or ‘counterintuitive’ conclusions is by subjecting one’s intuitions and emotional responses to effortful critical scrutiny. Another way is not to have these intuitions in the first place. Are utilitarians more ‘rational’ and calculating (Greene et al., 2004), or are they simply colder, and less averse to harming others (Bartels & Pizarro, 2011)?

Our results offer significant further support to the view that utilitarian judgments are often driven by lack of empathic concern and a diminished aversion to harming others. But they only offer a limited and qualified support for the view that such judgments might also be driven by explicit deliberation.

### 4.1 Cold morality: utilitarian judgment and lack of empathic concern

At the behavioural level, we found that increased rates of counterintuitive utilitarian judgment were associated both with ‘need for cognition’, a tendency to engage in effortful deliberation, and psychoticism, a personality trait associated with aggression and lack of empathic concern. Importantly, these two measures were statistically independent, suggesting that they relate to distinct factors driving utilitarian judgment.

In a previous study we reported that counterintuitive moral judgments in general, as well as counterintuitive utilitarian judgments in particular, were associated with increased activation in the SCC, a brain area implicated in empathic concern and guilt (Decety et al., 2012; Green et al. 2010; Moll et al. 2006; Zahn et al., 2009a, 2009b), compared to contrary ‘intuitive’ moral judgments (Kahane et al., 2012). By contrast, here we found that psychoticism was at once *positively* correlated with the number of utilitarian judgments and *negatively* correlated with SCC activation. This finding offers significant further support to the hypothesis that utilitarian judgment can be based in a deficit in empathic concern and social emotion even in healthy individuals, and suggests that the SCC reflects the degree of aversive response that individuals experience when they endorse acts that harm others in the context of making controversial utilitarian judgments.

Previous work has shown that individuals who score higher on the psychoticism scale are characterized by non-conformity to social norms, and by lack of emotionality and empathic concern, hostility, and aggression (Eysenck, 1976). Such individuals perceive media violence as more comical and enjoyable, and show rapid habituation to violent material (Bruggemann & Barry, 2002), and reduced aversion to killing enemies in a video game (Ravaja, Turpeinen, Saari, Puttonen, & Keltikangas-Jarvinen, 2008). Since the association between psychoticism and utilitarian judgment related only to moral dilemmas that typically involved causing extreme harm to innocents, it is likely that the observed association between psychoticism and utilitarian judgment reflected a reduced aversion (or even indifference) to harming others rather than a general nonconformist tendency.

This interpretation is strongly supported by recent research associating utilitarian judgment with weaker emotional responses to harming others (Moretto et al., 2009; Cushman et al., 2012) and lower rates of trait empathy (Choe & Min, 2011; Crockett et al., 2010), as well as with higher levels of testosterone (Carney & Mason, 2010). Utilitarian judgment has also been recently associated with psychopathy (Bartels & Pizarro, 2011; Glenn et al., 2010; Koenigs et al., 2012), a trait that is correlated with psychoticism (Hare, 1982; Corr, 2010). This growing body of evidence is also in line with the influential proposal that psychopathy is due to the malfunctioning of a ‘violence inhibition mechanism’ that is naturally operative in most humans (Blair, 1995).

Further research, however, is needed to clarify the precise relation between psychoticism, SCC activation, and diminished aversion to harming others. The decreased activation in the SCC observed here may underlie the set of dispositions associated with psychoticism, or it might be a consequence of a greater degree of psychoticism, a causal question that should be investigated using longitudinal studies. The relation between the SCC and aversion to harm also requires further investigation. It seems unlikely that the observed SCC activation reflects an immediate emotional aversion to the prospect of harming others, since we previously found that the SCC was associated with counterintuitive judgments endorsing such harm rather than with intuitive judgment opposing it (Kahane et al., 2012). This SCC activation is thus more likely to reflect the guilt most individuals experience when they *do* agree to harm others, and more generally, to reflect a negative emotional response to the violation of a common moral norm (in line with Green et al., 2010; Zahn et al., 2009a, 2009b). Finally, our findings suggest that damage to the SCC might be implicated in the increase in utilitarian judgment observed in patients with VMPFC damage (Ciaramelli et al., 2007; Koenigs et al., 2007; Moretto et al., 2009), a hypothesis that, again, needs further investigation.

Contrary to our prediction, psychoticism was not associated with lower ratings on a self-report measure of perceived difficulty. One possibility is that although it was in fact easier for individuals with a higher degree of psychoticism to make utilitarian judgments compared to other subjects, they nevertheless did not experience these judgments as subjectively easier. Another possibility is that participants higher in psychoticism were reluctant to report that they found it easy to endorse acts that directly harm innocents as others may see this in a negative light.

### 4.2 Calculating morality: utilitarian judgment and need for cognition

Counterintuitive utilitarian judgments have recently been associated with feeling guilty (Choe & Min, 2011), and in a previous study we reported that they were correlated with increased SCC activation, compared to contrary intuitive judgments (Kahane et al., 2012). These findings strongly suggest that although individuals with a higher degree of psychoticism (or psychopathy) arrive at counterintuitive utilitarian conclusions because of a deficit in empathic concern, many individuals nevertheless find such judgments aversive.

Some individuals thus need to effortfully overcome their natural aversion to harming others when making counterintuitive utilitarian judgments. It is a further question, however, whether explicit deliberation and moral reasoning play a distinctive role in utilitarian judgment even in these individuals. Our finding that ‘need for cognition’ was associated with greater rates of counterintuitive utilitarian judgments (yet not with counterintuitive deontological ones) is in line with this hypothesis (see also Bartels, 2008). However, as we previously reported, such utilitarian judgments were not generally associated with greater activation in the DLPFC or other brain areas implicated in deliberative processing (Kahane et al., 2012). Nor did we observe here an association between ‘need for cognition’ and DLPFC activation and other markers of effortful cognitive effort, such as perceived difficulty. This suggests that ‘need for cognition’ may lead to increased rates of counterintuitive utilitarian judgment not through greater engagement in explicit reasoning, but through some other factor. One possibility is that individuals who are more strongly disposed to effortful cognition also develop a general tendency to discount intuitive or emotional cues, and to be drawn to solutions to problems that appear more stereotypically calculating and ‘rational’ (for further discussion, see Kahane, 2012). This suggestion is in line with a recent study that found that a disposition to utilitarian solutions was associated, not with greater cognitive ability, but with a difficulty with reasoning thoughtfully about one’s emotions (Koven, 2011).

It is noteworthy that although ‘need for cognition’ was associated with greater rates of utilitarian judgments in dilemmas where such judgments were counterintuitive, it was *not* associated with greater rates of deontological judgments in dilemmas where *these* judgments were counterintuitive. However, since we found no evidence that the correlation between ‘need for cognition’ and counterintuitive utilitarian judgment reflects explicit moral reasoning, this finding appears to offer little support to the view that utilitarian judgment is preferentially associated with such explicit reasoning (Greene, 2008). Moreover, on this latter view one would expect that extreme counterintuitive deontological judgments (e.g. refusal to tell a white lie, however harmful the truth) should be driven by especially strong immediate emotional responses, predicting both that such judgments would be *negatively* correlated with ‘need for cognition’, and that they would be easier to make. Neither prediction is borne out by our findings. As previously reported, such counterintuitive deontological judgments were perceived as more difficult than contrary utilitarian ones (Kahane et al., 2012).

## 5 Conclusion

Utilitarianism has often been presented as the reasoned and systematic generalization of natural human empathic concern (Hare, 1981; Singer, 1979), and previous research has portrayed utilitarian judgment as an island of calm reason in a raging sea of unreflective emotional response—a rare example of genuine moral reasoning that can resist immediate gut reactions (Greene, 2008; Greene et al., 2004).

**Fig. 4:**
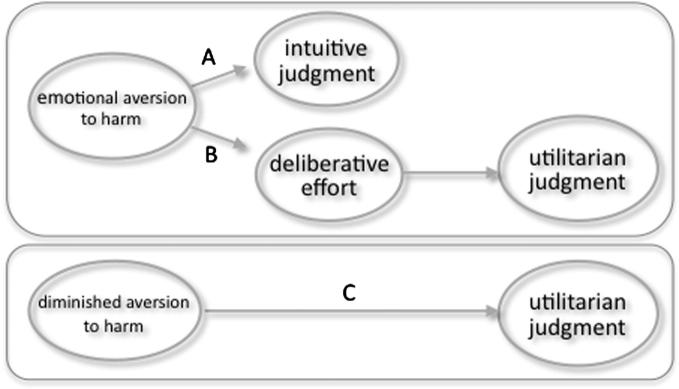
Dual pathway to counterintuitive utilitarian judgment. (A) Moral judgments in response to moral dilemmas are often based on an immediate intuition or affective response. (B) Previous research suggested that some individuals use deliberative effort to overcome this immediate response and reach counterintuitive utilitarian conclusions. (C) Such utilitarian judgments, however, might also be due to an absent or reduced affective response. Such a lack of empathic concern appears to drive utilitarian judgment in patients with damage to the ventromedial prefrontal cortex. Our results, coupled with other recent research, suggests that it also underlies utilitarian judgment in healthy individuals higher on ‘psychoticism’ or psychopathy.

Recent research has begun to cast doubt on any simple identification of utilitarian judgment with such explicit deliberation (Moretto, 2009; Bartels & Pizarro, 2011; Carney & Mason, 2010; Choe & Min, 2011; Crockett et al. 2010; Glenn et al., 2010; Koven, 2011; Koenings et al., 2012; Cushman et al., 2012). Our neuroimaging findings offer significant further evidence that counterintuitive utilitarian judgments are often driven, not by rational reflection or ‘generalized benevolence’ (Smart, 1961), but by a *deficit* in empathic concern and an indifference to harming others (see Fig. 4). One is reminded of John Stuart Mill’s remark on Jeremy Bentham, the founding father of utilitarianism: “[i]n many of the most natural and strongest feelings of human nature he had no sympathy” (Ryan, 1987).

Moreover, although a lack of aversion to harming might lead to greater rates of utilitarian judgment in certain contexts, such a disposition is highly problematic even from a utilitarian standpoint. Utilitarian thinkers often argue that a strong aversion to harming others is itself of great utility because, although it can lead to suboptimal choices in some situations, in the long run it would lead to better consequences than a cold and calculating attitude that is not constrained by such aversion (Sidgwick, 1907).

We do not want to be the slaves of our passions and intuitions. We admire the capacity to arrive at counterintuitive moral conclusions when it is based in a tendency to critically reflect on and, when appropriate, overcome common moral intuitions. The association between counterintuitive utilitarian judgement and a tendency to seek effortful cognitive activity can thus seem to favour such judgments (Greene, 2008). But the parallel association with lack of empathic concern and antisocial tendencies is rather less flattering (Bartels & Pizarro, 2011)—a tendency to endorse counterintuitive views is sinister when it merely reflects an indifference to harming others and a lack of responsiveness to common norms.

## Footnotes

2 The coldness in question refers to a lack of empathic concern and diminished prosocial emotion. It need not imply a general absence of emotion. Indeed, patients with VMPFC damage and psychopaths reject more unfair offers in the Ultimatum Game, a response pattern that is likely to be due to increased anger (Koenigs et al., 2007). In line with this, a recent study reports that a general disposition to feel angry was associated with greater rates of utilitarian judgment (Choe & Min, 2011).

3 Example of DI dilemma: “A runaway trolley is heading down the tracks toward five workmen who will be killed if the trolley proceeds on its present course. You are on a footbridge over the tracks, in between the approaching trolley and the five workmen. Next to you on this footbridge is a stranger who happens to be very large. The only way to save the lives of the five workmen is to push this stranger off the bridge and onto the tracks below where his large body will stop the trolley. The stranger will die if you do this, but the five workmen will be saved. Should you push the stranger onto the tracks in order to save the five workmen?”

4 Example of UI dilemma: “The husband of your close friend Jane told you that he had a secret romantic involvement with another woman four years ago but would never want to do so again, since his marriage means so much to him. On a night out with Jane she mentions that she was worried about her marriage four years ago. Jane goes on to say that her marriage is immensely important to her and that if her husband was ever unfaithful it would destroy the marriage because she would never be able to trust him again. She asks whether you’ve ever heard anything to suggest her husband isn’t faithful. Should you tell Jane that her husband had an affair?” For a complete list of dilemmas see Kahane et al. (2012).

## References

[b0010] Bartels D. (2008). Principled moral sentiment and the flexibility of moral judgment and decision making. Cognition.

[b0015] Bartels D.M., Pizarro D.A. (2011). The mismeasure of morals: Antisocial personality traits predict utilitarian responses to moral dilemmas. Cognition.

[b0025] Behrens T.E., Hunt L.T., Woolrich M.W., Rushworth M.F. (2008). Associative learning of social value. Nature.

[b0030] Blair R.J. (1995). A cognitive developmental approach to mortality: Investigating the psychopath. Cognition.

[b0035] Bruggemann J.M., Barry R.J. (2002). Eysenck’s P as a modulator of affective and electrodermal responses to violent and comic film. Personality and Individual Differences.

[b0045] Cacioppo J.T., Petty R.E., Feinstein J.A., Jarvis W.B.G. (1996). Dispositional differences in cognitive motivation: The life and times of individuals varying in need for cognition. Psychological Bulletin.

[b0050] Carney D.R., Mason M.F. (2010). Decision making and testosterone: When the ends justify the means. Journal of Experimental Social Psychology.

[b0055] Choe S.Y., Min K.-H. (2011). Who makes utilitarian judgments? The influences of emotions on utilitarian judgments. Judgment and Decision Making.

[b0060] Ciaramelli E., Muccioli M., Làdavas E., di Pellegrino G. (2007). Selective deficit in personal moral judgment following damage to ventromedial prefrontal cortex. Social Cognitive and Affective Neuroscience.

[b0065] Cima M., Tonnaer F., Hauser M.D. (2010). Psychopaths know right from wrong but don’t care. Social Cognitive and Affective Neuroscience.

[b0070] Corr P.J. (2010). The psychoticism–psychopathy continuum: A neuropsychological model of core deficits. Personality and Individual Differences.

[b0075] Crockett M.J., Clark L., Hauser M.D., Robbins T.W. (2010). Serotonin selectively influences moral judgment and behavior through effects on harm aversion. Proceedings of the National Academy of Sciences.

[b0085] Cushman F., Gray K., Gaffey A., Mendes W.B. (2012). Simulating murder: The aversion to harmful action. Emotion.

[b0080] Cushman F., Young L., Hauser M. (2006). The role of conscious reasoning and intuition in moral judgment: Testing three principles of harm. Psychological Science.

[b0090] Decety J., Michalska K.J., Kinzler K.D. (2012). The contribution of emotion and cognition to moral sensitivity: A neurodevelopmental study. Cerebral Cortex.

[b0095] Evans A.C., Collins D.L., Mills S.R., Brown E.D., Kelly R.L., Peters T.M. (1993). 3D statistical neuroanatomical models form 305 MRI volumes. Proceedings of IEEE-Nuclear Science Symposium and Medical Imaging.

[b0100] Eysenck H.J. (1976). Psychoticism as a dimension of personality.

[b0105] Eysenck H.J. (1992). The definition and measurement of psychoticism. Personality and Individual Differences.

[b0110] Eysenck H.J., Eysenck S.B.J. (1991). Eysenck personality questionnaire – revised (EPQ-R).

[b0115] Friston K.J., Ashburner J., Frith C.D., Poline J.B., Heather J.D., Frackowiak R.S. (1995). Spatial registration and normalization of images. Human Brain Mapping.

[b0125] Glenn A.L., Koleva S., Iyer R., Graham J., Ditto P.H. (2010). Moral identity in psychopathy. Judgment and Decision Making.

[b0120] Glenn A.L., Raine A., Schug R.A. (2009). The neural correlates of moral decision-making in psychopathy. Molecular Psychiatry.

[b0130] Green S., Lambon Ralph M.A., Moll J., Stamatakis E.A., Zhan R. (2010). Selective functional integration between anterior temporal and distinct fronto-mesolimbic regions during guilt and indignation. Neuroimage.

[b0140] Greene J.D., Morelli S.A., Lowenberg K., Nystrom L.E., Cohen J.D. (2008). Cognitive load selectively interferes with utilitarian moral judgment. Cognition.

[b0145] Greene J.D., Nystrom L.E., Engell A.D., Darley J.M., Cohen J.D. (2004). The neural bases of cognitive conflict and control in moral judgment. Neuron.

[b0135] Greene J.D., Sinnott-Armstrong W. (2008). The secret joke of Kant’s soul. Moral psychology: The neuroscience of morality.

[b0150] Greene J.D., Sommerville R.B., Nystrom L.E., Darley J.M., Cohen J.D. (2001). An fMRI investigation of emotional engagement in moral judgment. Science.

[b0165] Hare R.M. (1981). Moral thinking.

[b0160] Hare R.D. (1982). Psychopathy and the personality dimensions of psychoticism, extraversion and neuroticism. Personality and Individual Differences.

[b0170] Hazlitt, W. (1824/1991). *The spirit of the age*. In E. D. Mackerness (Ed.), Plymouth, pp. 19–35.

[b0315] Hermans E.J., Putman P., Van Honk J. (2006). Testosterone administration reduces empathetic behavior: A facial mimicry study. Psychoneuroendocrinology.

[b0320] Kahane G. (2012). On the wrong track: Process and content in moral psychology. Mind and Language.

[b0185] Kahane G., Shackel N. (2010). Methodological issues in the neuroscience of moral judgment. Mind and Language.

[b0190] Kahane G., Wiech K., Shackel N., Farias M., Savulescu J., Tracey I. (2012). The neural basis of intuitive and counterintuitive moral judgement. Social, Cognitive and Affective Neuroscience.

[b0205] Koenigs M., Kruepke M., Zeier J., Newman J.P. (2012). Utilitarian moral judgment in psychopathy. Social, Cognitive and Affective Neuroscience.

[b0200] Koenigs M., Young L., Adolphs R., Tranel D., Cushman F., Hauser M. (2007). Damage to the prefrontal cortex increases utilitarian moral judgements. Nature.

[b0210] Koven N.S. (2011). Specificity of meta-emotion effects on moral decision-making. Emotion.

[b0215] Krajbich I., Adolphs R., Tranel D., Denburg N.L., Camerer C.F. (2009). Economic games quantify diminished sense of guilt in patients with damage to the prefrontal cortex. Journal of Neuroscience.

[b0220] Mayberg H.S., Brannan S.K., Janet L.T., Silva J.A., Mahurin R.K., McGinnis S. (2000). Regional metabolic effects of fluoxetine in major depression: Serial changes and relationship to clinical response. Biological Psychiatry.

[b0225] Mendez M.F., Anderson E., Shapira J.S. (2005). An investigation of moral judgement in frontotemporal dementia. Cognitive and Behavioral Neurology.

[b0325] Moll J., de Oliveira-Souza R. (2007). Moral judgments, emotions and the utilitarian brain. Trends in Cognitive Sciences.

[b0240] Moll J., Krueger F., Zahn R., Pardini M., de Oliveira-Souza R., Grafman J. (2006). Human fronto–mesolimbic networks guide decisions about charitable donation. Proceedings of the National Academy of Sciences.

[b0245] Moretto G., Làdavas E., Mattioli F., di Pellegrino G. (2009). A psychophysiological investigation of moral judgment after ventromedial prefrontal damage. Journal of Cognitive Neuroscience.

[b0250] Nichols, T. E., 2008. *Re: Multiple regression: Testing an interaction at the second level. SPM email discussion list*. Retrieved <https://www.jiscmail.ac.uk/cgi-bin/wa.exe?A2=SPM;PPOtEw;20080318094435%2B0000>.

[b0260] Ravaja N., Turpeinen M., Saari T., Puttonen S., Keltikangas-Jarvinen L. (2008). The psychophysiology of James Bond: Phasic emotional responses to violent video game events. Emotion.

[b0270] Ryan, A. (Ed.), (1987). Utilitarianism and other essays: John Stuart Mill and Jeremy Bentham, London (pp. 132–175).

[b0280] Shine J., Hobson J. (1997). Construct validity of the Hare Psychopathy Checklist, Revised, on a UK prison population. Journal of Forensic Psychiatry and Psychology.

[b0285] Sidgwick H. (1907). The methods of ethics.

[b0290] Singer P. (2005). Ethics and intuitions. Journal of Ethics.

[b0295] Smart J.J.C. (1961). An outline of a system of utilitarian ethics.

[b0300] Unger P. (1996). Living high and letting die: Our illusion of innocence.

[b0310] Zahn R., de Oliveira-Souza R., Bramati I., Garrido G., Moll J. (2009). Subgenual cingulate activity reflects individual differences in empathic concern. Neuroscience Letters.

[b0305] Zahn R., Moll J., Paiva M., Garrido G., Krueger F., Huey E.D. (2009). The neural basis of human social values: Evidence from functional MRI. Cerebral Cortex.

