# Supplementary material for: Cold or calculating? Reduced activity in the subgenual cingulate cortex reflects decreased emotional aversion to harming in counterintuitive utilitarian judgment
Source: Cognition. 2013 Mar;126(3):364–72. doi: 10.1016/j.cognition.2012.11.002 (PMC3629560; doi:10.1016/j.cognition.2012.11.002)
Supplement: Supplementary data 1 [file mmc1.docx]

**Supplementary Material**

**for**

**Cold or Calculating?**

**Reduced activity in the subgenual cingulate cortex reflects decreased emotional aversion to harming in counterintuitive utilitarian judgment**

Katja Wiech*, Guy Kahane, Nicholas Shackel, Miguel Farias, Julian Savulescu, Irene Tracey

^*^To whom correspondence should be addressed:

[kwiech@fmrib.ox.ac.uk](mailto:kwiech@fmrib.ox.ac.uk)

Supplementary Figure 1

A B C


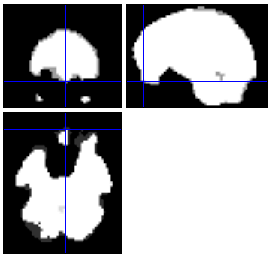

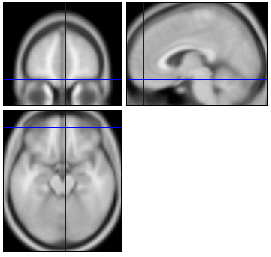

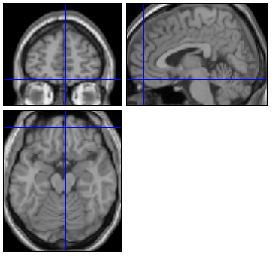


(A) Group implicit mask showing the coverage of ventral frontal brain regions, coregistered to the canonical T1 MNI152 brain (B) and the canonical T1 single subject brain provided by SPM5 (C).

Supplementary Figure 2


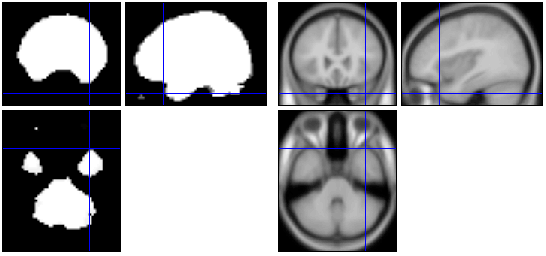

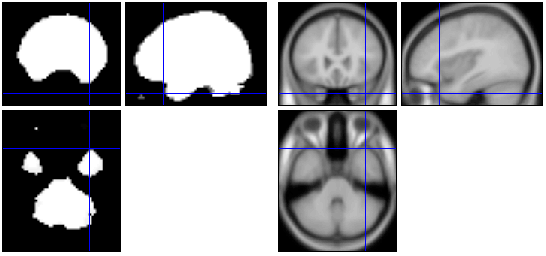

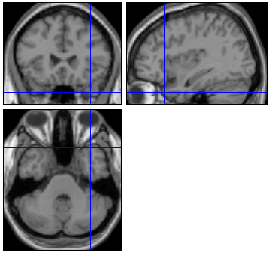


(A) Group implicit mask showing the coverage of anterior temporal brain regions, coregistered to the canonical T1 MNI152 brain (B) and the canonical T1 single subject brain provided by SPM5 (C).

**Supplementary Results**

**Table S1. Brain regions showing a negative correlation with psychoticism during counterintuitive utilitarian judgments**

| **brain region** | **laterality** | **Brodman area** | **MNI coordinates** | | | **cluster size**  **(voxel)** | **Z score** |
| --- | --- | --- | --- | --- | --- | --- | --- |
|  |  |  | x | y | z |  |  |
| visual cortex | R | 19 | 42 | -84 | 24 | 14 | 3.19 |
| SCC | R | 25 | 9 | 24 | 0 | 12 | 2.96 |
| SCC/OFC | R | 11/25 | 3 | 30 | -3 |  | 2.68 |
| precuneus | R | 30 | 15 | -48 | 15 | 13 | 2.70 |
| calcarine sulcus | R | 17 | 21 | -60 | 18 |  | 2.57 |

*Note:* R, right; SCC, subgenual cingulate cortex; OFC, orbitofrontal cortex; p< 0.01 uncorrected

at voxel level; minimum cluster extent: 5 contiguous voxels

**Table S2. Brain regions showing a negative correlation with ‘need for cognition’ during counterintuitive utilitarian judgments**

| **brain region** | **laterality** | **Brodman area** | **MNI coordinates** | | | **cluster size**  **(voxel)** | **Z score** |
| --- | --- | --- | --- | --- | --- | --- | --- |
|  |  |  | x | y | z |  |  |
| superior temporal lobe | R | 42 | 60 | -30 | -18 | 13 | 3.50 |
| inferior parietal lobe | L | 7 | -30 | -78 | 48 | 25 | 3.27 |
| OFC | R | 11 | 24 | 48 | 3 | 13 | 3.18 |
| cerebellum | L | - | -3 | -39 | -24 | 8 | 3.07 |
| cerebellum | R | - | 9 | -39 | -12 | 14 | 2.96 |

*Note:* L, left, R, right; OFC, orbitofrontal cortex; p< 0.01 uncorrected at voxel level;

minimum cluster extent: 5 contiguous voxels

**Table S3. Multiple regression analysis: Brain regions showing differential correlation with ‘need for cognition’ (‘NfC’) and psychoticism during counterintuitive utilitarian judgments**

| **brain region** | **laterality** | **Brodman area** | **MNI coordinates** | | | **cluster size**  **(voxel)** | **Z score** |
| --- | --- | --- | --- | --- | --- | --- | --- |
|  |  |  | x | y | z |  |  |
| ***NfC > psychoticism*** |  |  |  |  |  |  |  |
| visual cortex | R | 19 | 42 | -84 | 24 | 9 | 3.04 |
| SCC | R | 25 | 6 | 24 | -3 | 7 | 2.94 |
| precuneus | R | 17 | 21 | -54 | 15 | 20 | 2.82 |
| precuneus | R | 30 | 15 | -48 | 15 |  | 2.77 |
|  |  |  |  |  |  |  |  |
| ***psychoticism > NfC*** |  |  |  |  |  |  |  |
| primary motor cortex | R | 4 | 33 | -27 | 72 | 34 | 3.61 |
| primary motor cortex | R | 4 | 39 | -21 | 69 |  | 3.40 |
| premotor cortex | R | 6 | 27 | -18 | 78 |  | 2.53 |
| prem. cortex/VLPFC | R | 44 | 45 | 3 | 30 | 5 | 3.43 |
| cerebellum | L | - | -15 | -45 | -21 | 10 | 3.10 |
| premotor cortex | L | 6 | -9 | -18 | 81 | 5 | 2.69 |
| hippocampus | L | 20 | -39 | -12 | -15 | 6 | 2.62 |
| vermis | R | - | 6 | -75 | -15 | 8 | 2.53 |

*Note:* L, left, R, right; SCC, subgenual cingulate cortex; VLPFC, ventrolateral prefrontal cortex; p< 0.01 uncorrected at voxel level; minimum cluster extent: 5 contiguous voxels
